# Knowledge base dataset on nature-based solutions for wastewater treatment and stormwater management

**DOI:** 10.1016/j.dib.2025.111469

**Published:** 2025-03-15

**Authors:** Josep Pueyo-Ros, Massimiliano Riva, Gisela Gonzalvo-Henry, Sophie Guillaume-Ruty, Joaquim Comas

**Affiliations:** a Catalan Institute for Water Research (ICRA-CERCA), Spain; b University of Girona, Spain; c Research Unit REVERSAAL, INRAE, France; d LEQUIA, University of Girona, Spain

**Keywords:** Nature-based solutions, Wastewater, Stormwater, Natural treatment systems, SUDS

## Abstract

We introduce a knowledge base on Nature-Based Solutions (NBS) for wastewater treatment and stormwater management. The knowledge base includes (i) a catalogue of solutions and their variables, such as type of water that they can manage, ecosystem services provided, and operational constraints; (ii) a dataset of scientific publications related to each of the solutions included in the catalogue; and, (iii) a dataset of monitoring samples collected on treatment performance of NBS for wastewater treatment, including biological oxygen demand, chemical oxygen demand, ammonia, nitrate, total nitrogen, phosphate and pathogens (Escherichia Coli and Helminth eggs). Data collection methods employed to build the knowledge base included elicitation workshops, expert assessments, literature review and experimental data. A notable inclusion is the database of scientific publications, boasting 513 entries and providing a dynamic reference point for solution insights. Users can actively contribute to the database, ensuring its continuous enrichment and relevance. Whereas the goal of the knowledge base is to feed the algorithms developed in the Nat4Wat tool, this data can be used for several purposes, from modelling surface requirements for different NBS to being used as a sandbox for data science students. All the data can be accessed directly using a REST API of the Nat4Wat tool, which downloads the last version of the different datasets; or can be downloaded from a static repository where versions are regularly updated.

Specifications Table

**Table:** 

Subject	Water Science and Technology.
Specific subject area	Nature-based solutions for wastewater treatment and stormwater management.
Type of data	
Data collection	The knowledge base was built using expert workshops, literature reviews, and experimental data. The NBS catalogue development included: (1) upgrading the SNAPP project legacy, which defined major NBS types and sub-types via workshops using the IDEA protocol; (2) integrating Enhanced Natural Treatment Systems (ENTS) from MULTISOURCE pilots using data consensus workshops; (3) adding stormwater management (SWM) solutions via grey literature and expert input. Scientific publications were collected in two stages: SNAPP (2019) and MULTISOURCE (2020–2022), covering wastewater and SWM solutions through curated database searches.
Data source location	Please mention where the data were collected (e.g. geographical coordinates) or where the data are stored (typically your affiliation). Data were collected in the SANNAT and MULTISOURCE projects.
Data accessibility	Please note: All raw data referred to in this article must be made publicly available in a data repository prior to publication. Please indicate here where your data are hosted (the URL must be working at the time of submission and editors and reviewers must have anonymous access to the repository): Repository name: Zenodo Data identification number: 10.5281/zenodo.13885202 Direct URL to data: https://doi.org/10.5281/zenodo.13885202 The database [6] can be downloaded directly from Zenodo using the DOI provided above. Alternatively, a REST API gives access to the most recent version used in the Nat4Wat tool. The query https://nat4wat-api.icradev.cat/technologies/technologies gives access to the catalogue (record-oriented JSON format), the query https://nat4wat-api.icradev.cat/sci-studies/sci-publications gives access to the scientific publications (record-oriented JSON format), and the query https://nat4wat-api.icradev.cat/sci-studies/sci-studies gives access to the treatment details for scientific publications related to solutions for wastewater treatment (column-oriented JSON format), adding the parameter (records=true) returns the same dataset but record-oriented (https://snappapi-v2.icradev.cat/sci-studies/sci-publications?records=true).
Related research article	None

## 1 Value of the Data

• We posit that making this database accessible in accordance with the FAIR principles can prove beneficial to scholars engaged in research pertaining to nature-based solutions, water management, or even data science in general.
• This dataset is valuable because it provides a comprehensive and structured resource on Nature-Based Solutions (NBS) for wastewater and stormwater management. It combines expert knowledge, scientific literature, and real-world performance data, offering insights into various NBS types, their benefits, and operational constraints. The inclusion of 513 scientific publications and detailed monitoring data enhances its credibility and practical relevance. Users can leverage it for designing, modeling, and optimizing NBS for diverse socio-environmental contexts. Additionally, its accessibility via API and static repository ensures usability across research, policy, and education, promoting sustainable water management practices worldwide.
• For instance, in the Nat4Wat tool, a decision-support system about nature-based solutions for water management, the dataset is utilized to identify the most suitable solution for a given scenario, which considers the specific water requirements and the user's objectives. The data is employed to exclude unsuitable solutions, to calculate the requisite surface area for water management, and to conduct a multicriteria decision analysis to guide the user towards the optimal solution, providing an assessment based on different environmental, economic and social attributes of each solution. The same dataset can be used for other research or innovation projects dealing with nature-based solutions for wastewater treatment.
• Other potential applications include the education and training of data science students, as the knowledge base can be easily exported to a relational database to facilitate the development of SQL skills. Furthermore, the database contains sufficient information to facilitate the training of data pipeline skills, encompassing the processes of data curation and validation and modeling exercises such as surface estimation and outflow concentration estimation.

## 2 Background

The knowledge base presented here is part of the MULTISOURCE EU project. The goal of the project is to facilitate the systematic, citywide planning of nature-based solutions for urban water treatment, storage, and reuse. Among the tools created within MULTISOURCE, the Nat4Wat tool (https://nat4wat.icradev.cat) serves as a decision-support system, aiding users in choosing the most suitable nature-based solutions (NBS) for a specific context, which encompasses inflow conditions, outflow requirements, space availability, and other considerations such as the provision of specific ecosystem services or the skills required to operate the solution.

All algorithms executed by the tool are fuelled by three types of data: data obtained from experts, from literature and experimental data. The expert-based data is a catalogue of NBS for wastewater treatment and stormwater management. Moreover, the data obtained from literature is divided into two datasets. The first dataset contains scientific publications focused on one or several NBS included in the catalogue. The second dataset contains additional data about the monitoring samples mentioned in the publications, such as inflow, pollutant concentrations, surface area, etc. This dataset is specifically for publications about natural treatment systems, it does not contain information about stormwater management solutions.

## 3 Data Description

### 3.1 Catalogue of NBS for wastewater treatment and stormwater management

The catalogue comprises 54 solutions, 35 of which are dedicated to wastewater treatment (WWT) for various wastewater types, including combined sewer overflow (CSO) discharges, and 19 for stormwater management (SWM), encompassing rainwater and runoff water. The data was presented in tabular form, with details for each solution provided across 61 variables. Of those, 28 are common to all solutions, while 26 are specific to wastewater treatment solutions and 7 are specific to SWM solutions. A dataset, entitled 'description_catalog.csv', is stored within the database and contains the descriptions of the variables included in the catalogue. This description is also available as a web-based table: https://nat4wat-api.icradev.cat/reference_manual.html#tbl-vars-description. In this dataset, a value of '1′ (active) indicates that a solution is suitable for a specific type of water or for the removal of a particular pollutant. Conversely, a value of '0′ (inactive) signifies that a solution is unsuitable for that particular type of water or pollutant. Finally, a value of '2′ (not ideal) implies that a solution could potentially be suitable, but that it has not been designed or optimized for that specific purpose.

**Fig. 1:**
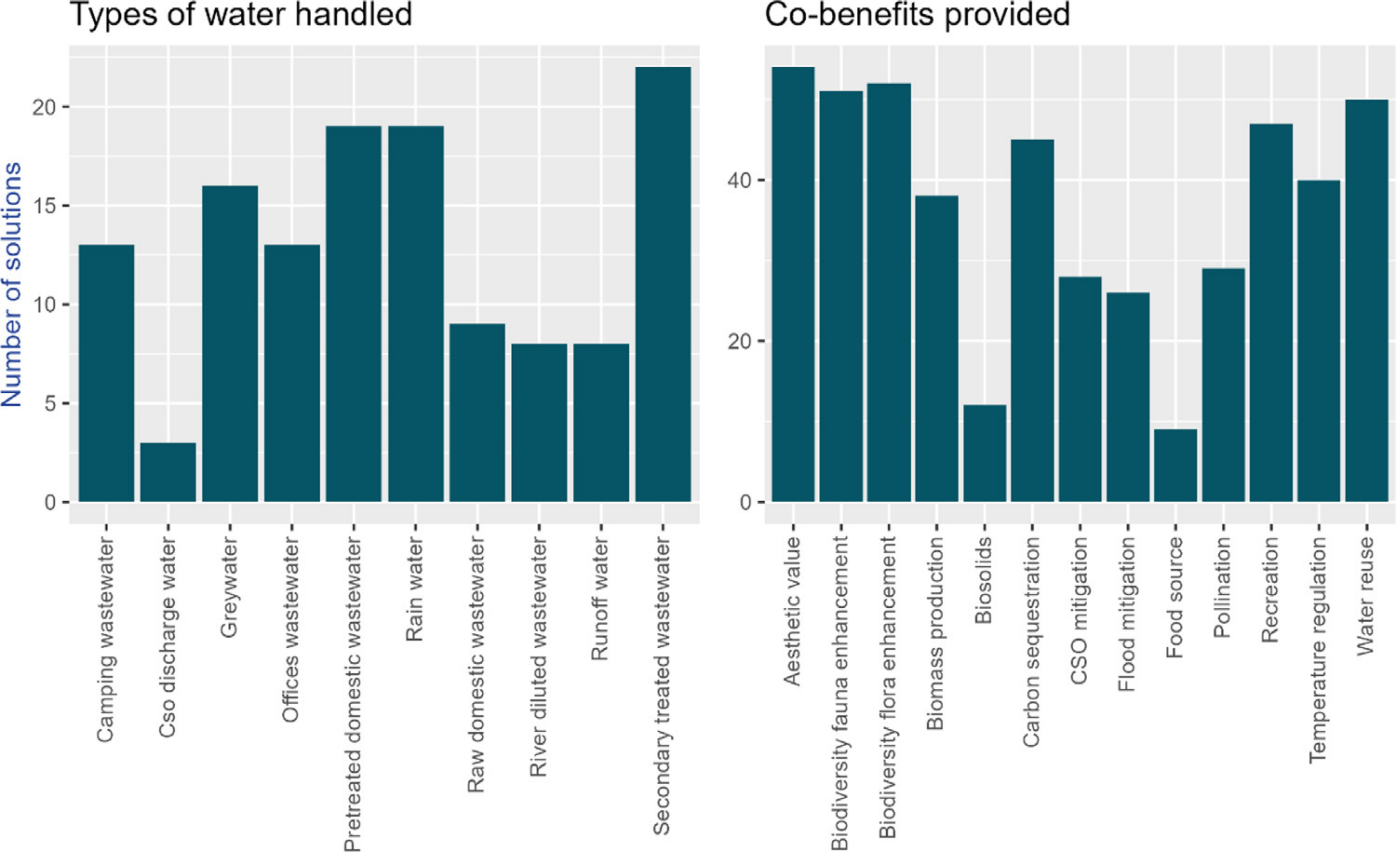
Types of water handled by the NBS (left) and cobenefits provided (right).

The solutions included in the knowledge base are designed to address all water types considered in the Nat4Wat tool. Additionally, they offer a diverse range of co-benefits in the form of ecosystem services (Fig. 1). Some co-benefits are not applicable to all solutions, as they represent the primary objectives of specific solutions. For instance, flood mitigation and CSO mitigation are considered co-benefits only for wastewater treatment solutions, as these outcomes are the primary goals of SWM solutions. Including them as co-benefits across all solutions could lead to double-counting in subsequent analyses, such as multicriteria decision analysis.

**Fig. 2:**
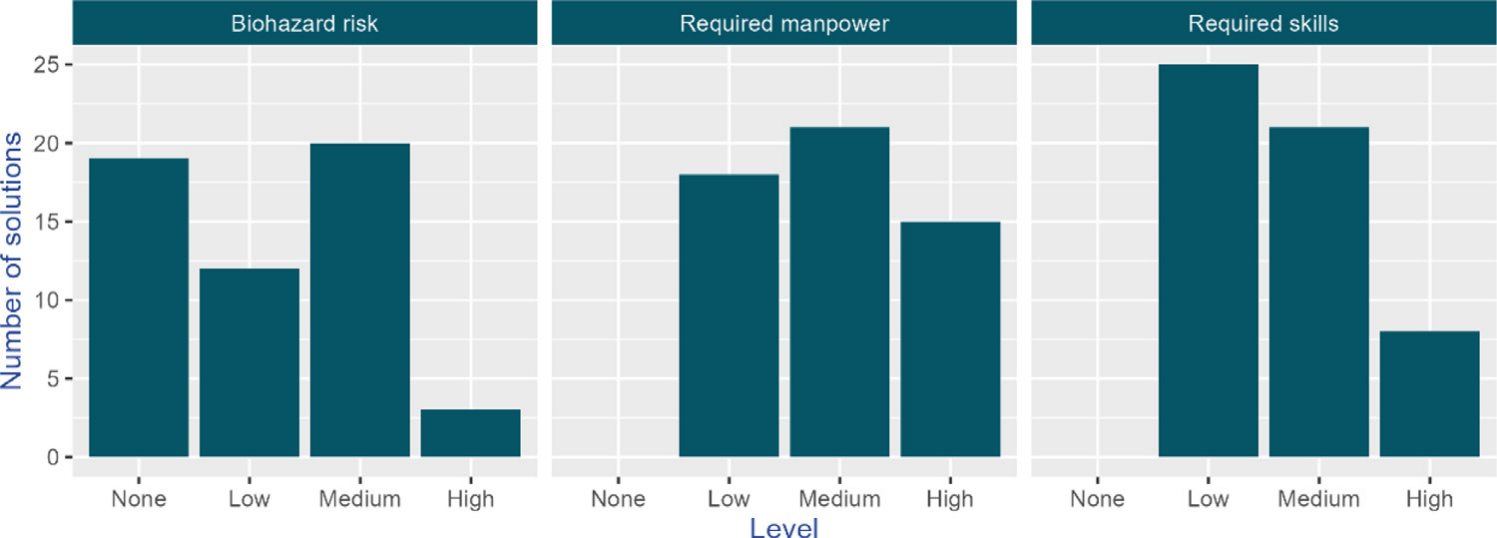
Barriers to the implementation of solutions in the knowledge base.

The knowledge base identifies three key barriers to the implementation: biohazard risk, required manpower, and necessary technical skills. They are categorized in a qualitative scale from 0 (none) to three (high), derived from expert judgment as explained in the methods section. Biohazard risk is a significant concern associated with these solutions, referring to the exposure to hazardous biological materials. The required manpower highlights the labor intensity needed to operate the solution, while the technical skills denote the level of training required for their effective use. Notably, only a small number of solutions pose a considerable biohazard risk, while most of them require only minimal technical skills (Fig. 2).

**Fig. 3:**
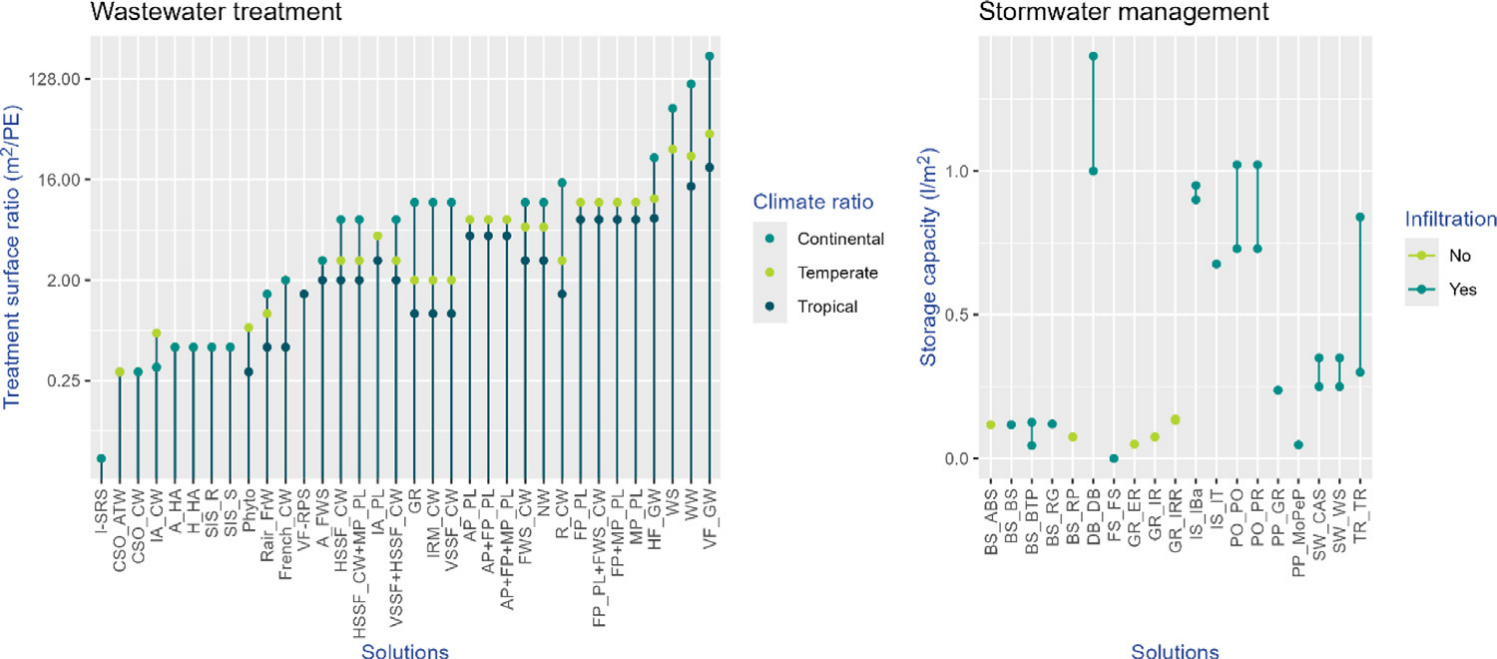
Expert-based data used to estimate the required surface for wastewater treatment (left) and stormwater management (right) solutions. Note that the scale in left panel is in log2. The acronyms description can be found in the catalogue of solutions.

The knowledge base also provides values, derived from expert assessments, to estimate the required surface area for a given water scenario, considering the climatic context for treatment performance (as shown in Fig. 3). These values are expressed as m^2^/person equivalent, but also translated to organic loading rates in grams of Biological Oxygen Demand (gr_BOD_/m^2^), assuming a production of 60 gr of BOD per person and day. Concerning SWM solutions, the storage capacity (l/m^2^) is calculated multiplying the depth by the porosity. It is also considered whether they can infiltrate water or only retain it (Fig. 3).

### 3.2 Scientific publications

**Fig. 4:**
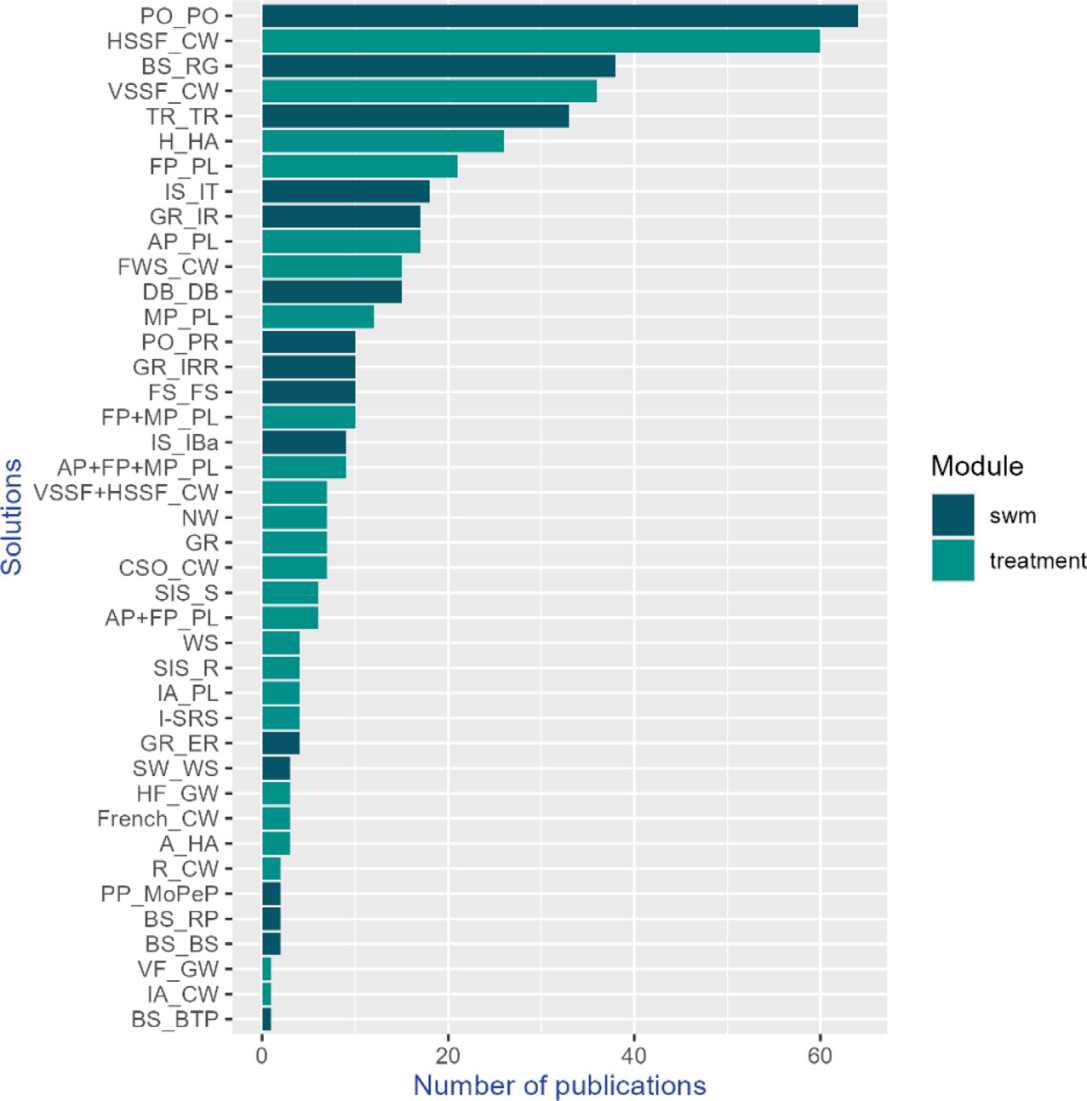
Number of scientific publications per solution.

The database of scientific publications comprises 513 publications related to the solutions in the knowledge base. Among these, 275 publications are dedicated to solutions for wastewater treatment, while 258 focus on solutions for stormwater management (SWM). Ponds for stormwater management are the solution with the highest number of publications, followed by constructed wetlands with horizontal flow (HSSF_CW) (Fig. 4).

It is noteworthy that 13 solutions lack corresponding scientific publications. Six of the solutions are linked with six out of the seven MULTISOURCE wastewater treatment pilot projects. It is anticipated that these innovative solutions will be published potentially after the MULTISOURCE project. The remaining missing solutions are as follows: facultative pond with free water surface wetland, horizontal flow treatment wetland with a maturation pond, and reactive media in a treatment wetland for wastewater treatment (3); anaerobic bioretention system, conveyance and attenuation swale, and grass reinforcement for stormwater management (3); and aerated treatment wetland for CSO for the treatment of combined sewer overflow discharges (1).

The structure of the database encompasses all necessary information for identifying and retrieving publications, including title, DOI, year of publication, authors, journal, issue, and pages. Users of the Nat4Wat tool are able to update the database with new publications, and for traceability, each publication includes details about the uploading user: username, email, and company (which is not publicly available). A field named ‘validation_status’ indicates whether a publication has been accepted after peer review, with only accepted publications being publicly available.

The monitoring samples dataset is a separate table within the database. Information is stored with data for modelling the surface in wastewater treatment solutions. Both datasets are linked using a common ID. A scientific publication may contain data from various sampling campaigns or different solutions. In such cases, each campaign is treated as a distinct observation, represented by a separate row in this dataset. The information in the monitoring samples dataset includes details about water characteristics (inflow rate, pollutant concentration, type of water, temperature) and solution attributes (surface, urban water system, people served, and year of operation). A metadata file, entitled ‘description_sci_publications_treatment_details.csv’, contains descriptions and units of each variable included. This data can also be uploaded by the users of the tool, and it is published after acceptance by peer-review.

**Fig. 5:**
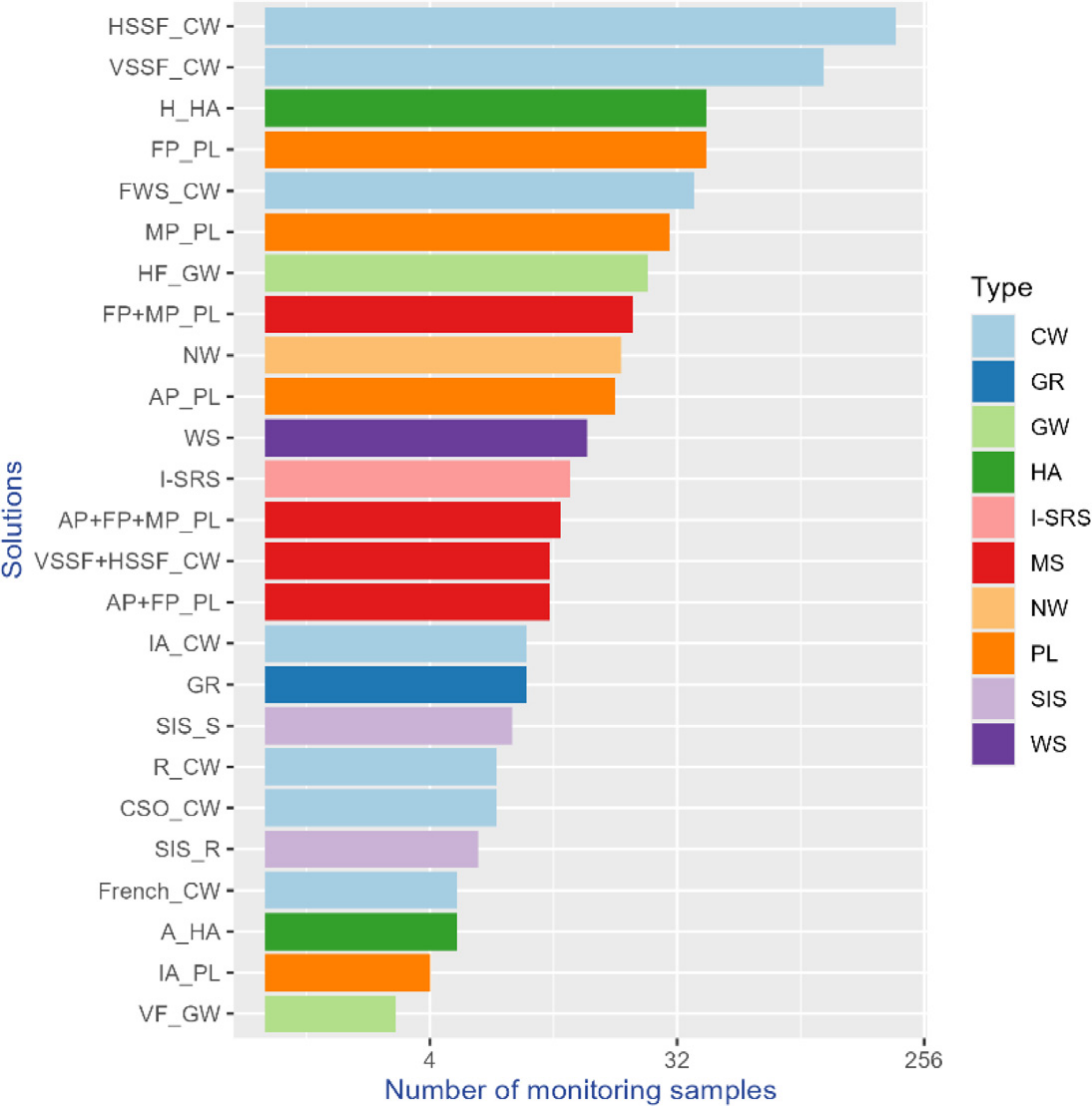
Number of monitoring samples by solution with information on treatment performance.

Fig. 5 shows the number of monitoring samples by solution. The monitoring samples dataset encompasses 672 sampling campaigns sourced from 199 publications. Constructed wetlands, also named treatment wetlands [5], particularly horizontal and vertical flow wetlands, showcase the highest number of samples, with hydroponic systems and facultative ponds also contributing significantly. As mentioned earlier, the six pilots from MULTISOURCE and three other solutions for wastewater treatment lack related publications and, consequently, do not appear in this dataset detailing treatment performance (Fig. 5). However, this data will be added from the experimental data collected in the pilots once validated within the project lifetime.

Moreover, it should be noted that this knowledge base is not static. It is a crowdsourced database, whereby researchers and technology providers can share their publications and monitoring samples. Additionally, the Nat4Wat tool incorporates data from market cases (i.e., real-world implementations of the solutions included in the catalogue), which complement the data presented in this data note.

Despite the absence of certain data points in the datasets presented in this paper (see limitations section), they may prove useful for a multitude of applications. In the Nat4Wat tool, the aforementioned information is utilized to identify the most suitable solution for a given scenario, taking into account the specific water requirements and the user's objectives. The data is used to exclude unsuitable solutions and to determine the required surface area for water management, and to conduct a multicriteria decision analysis to guide the user towards the optimal solution. However, other potential applications include the education and training of data science students, as the knowledge base can be readily exported to a relational database to facilitate the development of SQL skills. Furthermore, the database provides enough information to support the development of data processing and management skills, including data organization, validation, and quality control (e.g. [3]) and modelling exercises such as surface estimation and outflow concentration estimation. In conclusion, we posit that making this database accessible in accordance with the FAIR principles can prove beneficial to scholars engaged in research pertaining to nature-based solutions, water management, or even data science in general.

## 4 Experimental Design, Materials and Methods

The sources of data used to build the knowledge base were divided into expert-based knowledge, literature review and experimental data.

### 4.1 Catalogue of nature-based solutions

The construction of the dataset containing the NBS and their variables involved three distinct stages, delineated as follows:

1. Upgrade of the legacy from the SNAPP Project: The initial stage of the knowledge base development is inherited from the SNAPP project “Guidance on evidence-based practices for improved sanitation, water security and ecological health, with a focus on nature-based solutions”. This international project was funded by the National Center for Ecological Analysis and Synthesis (NCEAS, UC Santa Barbara) and involved participants across the globe, from Asia to America. More information in Cross et al. [2].
2. Addition of enhanced natural treatment systems (ENTS) from MULTISOURCE Pilots: The second stage incorporates information from the MULTISOURCE pilots, expanding the scope of the knowledge base by including enhanced natural treatment systems.
3. Inclusion of Nature-Based Solutions for Stormwater Management: The third and final stage integrates nature-based solutions specifically tailored for stormwater management into the knowledge base.

The SNAPP project played a pivotal role in establishing the foundation for the knowledge base within MULTISOURCE. The development of this knowledge base occurred through a series of elicitation workshops, primarily employing the IDEA protocol [4] – a modified Delphi method. The workshops, conducted in Santa Barbara (22-24 May 2018), Vienna (Feb 11th, 2019) and Girona (Nov 8th, 2019), aimed to harness expert knowledge on the most suitable Nature-Based Solutions (NBS) for wastewater treatment for diverse socio-environmental contexts and needs. These workshops involved more than 40 experts worldwide, covering international representation (America, Europe, Asia, and Africa) and expertise in all types of NBS. More info about the participants can be found in Acuña et al. [1]. Firstly, they collaboratively identified nine major types of NBS and 26 associated sub-types, encompassing different varieties and combinations. Subsequently, participants addressed several key aspects to define the catalogue.

The second stage in building the knowledge base involved the inclusion of the innovative NBS (or ENTS) studied in the MULTISOURCE pilots into the roster of available solutions. The gathering of the data and knowledge for this integration took place during a workshop held at the second annual meeting of the MULTISOURCE project in Leipzig on June 5th, 2023, attended by 23 NBS researchers, 4 public administrators, 4 technology providers, and 7 social scientists, from Europe, America and Asia. The workshop consisted of two rounds. In the initial round, all project partners were tasked with providing data for all pilots, along with indicating their confidence level in supplying this information. This approach mirrored the knowledge base structure established during the SNAPP project. In the second round, MULTISOURCE partners were organized by pilots, collaborating to achieve consensus on the data for their respective pilot projects (i.e. responsible partners for one pilot worked out consensus for only the ENTS of that pilot). All collected data were summarized and weighted based on the confidence levels reported by users. The workshop aimed to reach a consensus decision, ensuring the integration of pilot data into the knowledge base.

The SNAPP project did not encompass solutions for SWM. Therefore, the first step in including SWM was to establish a structured framework for the knowledge base. All existing variables related to co-benefits and barriers in the knowledge base were retained, and new values concerning the stormwater inputs were collaboratively defined with MULTISOURCE experts. The data collection for SWM solutions primarily relied on grey literature combined with expert knowledge. Desk work, as opposed to elicitation workshops, was the primary method for data collection of stormwater solutions. The main information source was Woods Ballard et al. [8] by CIRIA.

### 4.2 Scientific publications

Similar to the catalogue, the collection of scientific publications underwent a two-stage process. The initial batch of publications was collected during the SNAPP project, focusing solely on wastewater treatment solutions appearing on scientific journal papers (until 2019). Subsequently, within the MULTISOURCE project, the database was updated to include new scientific publications for wastewater treatment (2020-2022), data from grey literature (mainly design handbooks and databases with experimental data from project partners) and expanded to encompass SWM solutions.

In the first stage, scientific studies pertaining to the utilization of Nature-Based Solutions (NBS) in domestic wastewater systems constituted the first stage of publication collection. Using the Web-of-Science database, a comprehensive search was conducted on May 23rd, 2019, encompassing studies published in any year. Nine distinct searches, one for each type of NBS, were executed, with search terms strategically chosen to ensure the inclusion of all potentially relevant studies. Eligibility criteria were established, requiring full-text availability, English language, exclusion of laboratory case studies, prioritization of full-scale or pilot-scale studies, exclusion of reviews, and the inclusion of articles providing information on at least three selected variables characterizing NBS (more info in [1]).

Upon identifying potentially relevant articles, a meticulous selection process ensued. To ensure consistent choices, 30 % of the articles underwent a dual review (title and abstract) by two researchers. The final inclusion in the database was contingent on meeting the eligibility criteria outlined above.

During the abstract review, reviewers introduced additional information for each case study in the spreadsheet. These variables, organized into six domain removal efficiencies, water sources/types, sustainability indicators, co-benefits, elements of the urban wastewater system, and design and operational settings (see Fig. 2)—were filled in by extracting information from the abstract or, if necessary, by downloading and reviewing the full text of the article.

This information was divided into two datasets: a dataset of scientific publications, only storing the information related to the publication; and a second dataset of monitoring samples, storing the information on treatment performance retrieved from the publications and from the MULTISOURCE ENTS.

Then, in the second stage, the same methodological approach utilized during the SNAPP project was applied to update the database for wastewater treatment solutions, extending the data collection until the year 2022. In the context of stormwater management solutions, the objective was to compile a list of pertinent publications for each solution, facilitating future reference for tool users. The Nat4Wat tool does not rely on data-driven models to estimate the surface of SWM solutions, as it does for WWT solutions. Consequently, no additional data was extracted from the gathered publications. The search strategy was to gather relevant publications found on the SCOPUS database. While the 2019 search for papers on WWT solutions was conducted using the Web of Science database, we chose SCOPUS for this new search due to its broader coverage, according to recent studies [7]. Specific queries were crafted for each solution in the catalogue, combining the solution name with “runoff” or “stormwater” in the title or abstract. Consequently, the following queries were used:

• (TITLE-ABS-KEY ("Anaerobic bioretention system") AND (TITLE-ABS-KEY ("stormwater") OR TITLE-ABS-KEY ("runoff")))
• (TITLE-ABS-KEY ("Bioretention swale (or trench)") AND (TITLE-ABS-KEY ("stormwater") OR TITLE-ABS-KEY ("runoff")))
• (TITLE-ABS-KEY ("Bioretention tree pit") AND (TITLE-ABS-KEY ("stormwater") OR TITLE-ABS-KEY ("runoff")))
• (TITLE-ABS-KEY ("Conveyance and attenuation swale") AND (TITLE-ABS-KEY ("stormwater") OR TITLE-ABS-KEY ("runoff")))
• (TITLE-ABS-KEY ("Detention basin") AND (TITLE-ABS-KEY ("stormwater") OR TITLE-ABS-KEY ("runoff")))
• (TITLE-ABS-KEY ("Extensive green roof") AND (TITLE-ABS-KEY ("stormwater") OR TITLE-ABS-KEY ("runoff")))
• (TITLE-ABS-KEY ("Filter strips") AND (TITLE-ABS-KEY ("stormwater") OR TITLE-ABS-KEY ("runoff")))
• (TITLE-ABS-KEY ("Grass reinforcement") AND (TITLE-ABS-KEY ("stormwater") OR TITLE-ABS-KEY ("runoff")))
• (TITLE-ABS-KEY ("Infiltration basin") AND (TITLE-ABS-KEY ("stormwater") OR TITLE-ABS-KEY ("runoff")))
• (TITLE-ABS-KEY ("Infiltration trench") AND (TITLE-ABS-KEY ("stormwater") OR TITLE-ABS-KEY ("runoff")))
• (TITLE-ABS-KEY ("Intensive green roof") AND (TITLE-ABS-KEY ("stormwater") OR TITLE-ABS-KEY ("runoff")))
• (TITLE-ABS-KEY ("Intensive green roof with retention layer") AND (TITLE-ABS-KEY ("stormwater") OR TITLE-ABS-KEY ("runoff")))
• (TITLE-ABS-KEY ("Modular permeable paving") AND (TITLE-ABS-KEY ("stormwater") OR TITLE-ABS-KEY ("runoff")))
• (TITLE-ABS-KEY ("Pond") AND (TITLE-ABS-KEY ("stormwater") OR TITLE-ABS-KEY ("runoff")))
• (TITLE-ABS-KEY ("Pond retrofits") AND (TITLE-ABS-KEY ("stormwater") OR TITLE-ABS-KEY ("runoff")))
• (TITLE-ABS-KEY ("Rain garden") AND (TITLE-ABS-KEY ("stormwater") OR TITLE-ABS-KEY ("runoff")))
• (TITLE-ABS-KEY ("Raised planter") AND (TITLE-ABS-KEY ("stormwater") OR TITLE-ABS-KEY ("runoff")))
• (TITLE-ABS-KEY ("Trees") AND (TITLE-ABS-KEY ("stormwater") OR TITLE-ABS-KEY ("runoff")))
• (TITLE-ABS-KEY ("Wet swale") AND (TITLE-ABS-KEY ("stormwater") OR TITLE-ABS-KEY ("runoff")))

The aim was not to conduct an exhaustive review, but rather to identify pertinent publications for each solution included in the catalogue. If no publications were identified for certain solutions using the aforementioned queries, a manual search utilizing synonyms was conducted. Subsequently, the list of obtained references underwent a meticulous curation process, whereby publications that were not directly pertinent to the solutions or their capacity to manage stormwater were excluded. This curation process was based on a thorough examination of the titles and abstracts. By adopting this focused approach, it was possible to ensure that the final list of publications for SWM solutions remained both relevant and directly applicable for user consultation within the Nat4Wat tool.

## Limitations

**Fig. 6:**
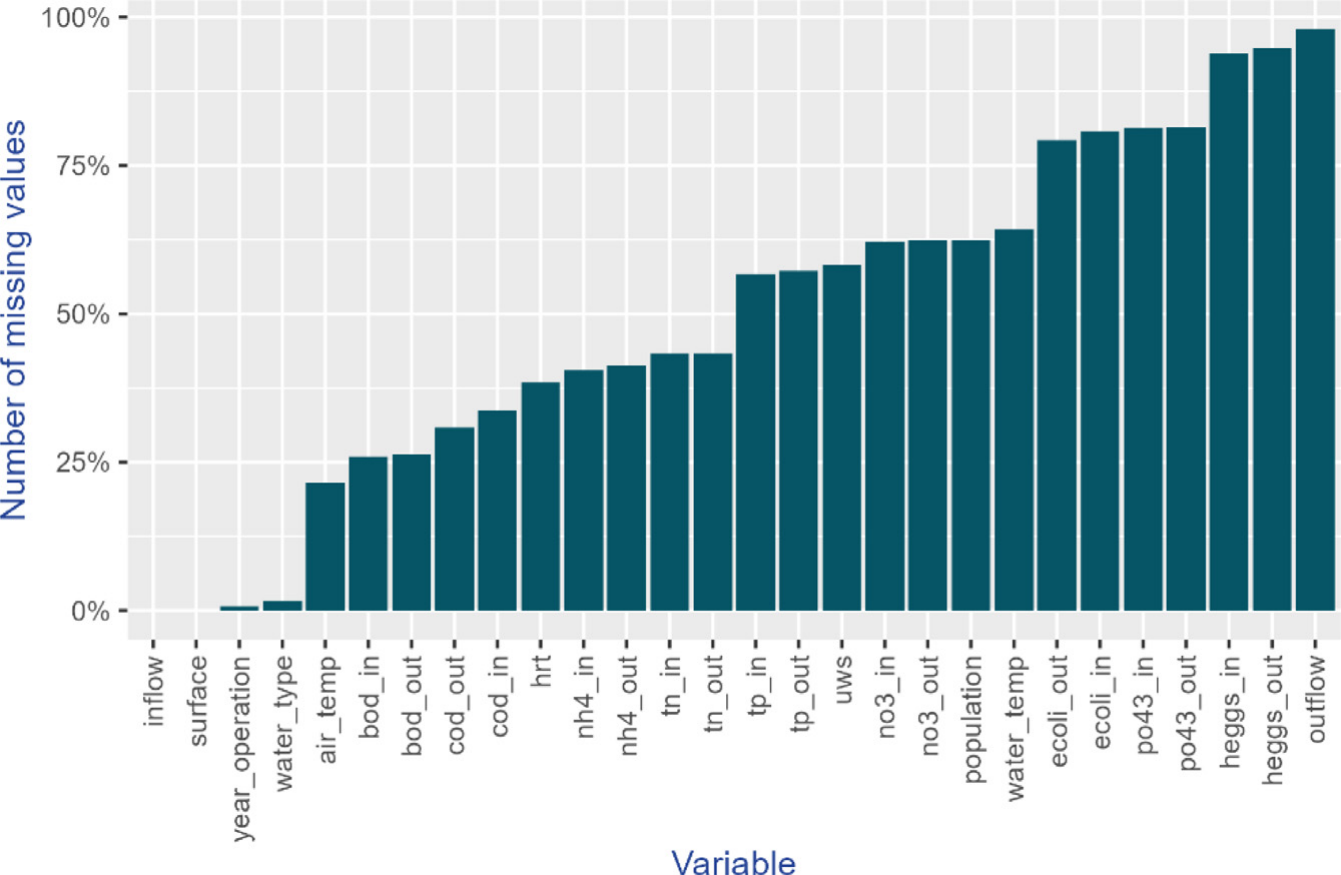
Number of missing values for each variable in the dataset.

Regarding performance data collected from scientific publications, not all information was consistently available in every scientific publication, resulting in varying numbers of missing values in the dataset. Consequently, certain analyses can only be conducted using a subset of the observations. Fig. 6 shows the number of missing values for each variable in the dataset. Outflow rate has the highest number of missing values because it was a variable added in a recent version of the dataset. Apart from that, the variables with the highest number of missing values are the count of Helminth eggs, followed by concentrations of E. coli. In contrast, the dataset is most complete for BOD concentrations, followed by Chemical Oxygen Demand (COD) and ammonia concentrations. Both inflow rate and surface variables have no missing values, as their availability was a prerequisite for inclusion in the dataset. Similarly, the year of operation has no missing values, because when information was absent in the paper, the year of publication was used instead (Fig. 6).

## Ethics Statement

The work presented here involved human subjects participating in the elicitation workshops. All subjects participated voluntarily and signed an informed consent. No personal data was collected during the workshops.

## CRediT authorship contribution statement

**Josep Pueyo-Ros:** Conceptualization, Methodology, Validation, Investigation, Data curation, Writing – original draft, Writing – review & editing, Visualization, Supervision. **Massimiliano Riva:** Investigation, Data curation, Writing – review & editing. **Gisela Gonzalvo-Henry:** Investigation, Data curation, Writing – review & editing. **Sophie Guillaume-Ruty:** Investigation, Data curation, Writing – review & editing. **Joaquim Comas:** Conceptualization, Methodology, Validation, Writing – review & editing, Supervision, Project administration, Funding acquisition.

## Data Availability

ZenodoKnowledge base for NBS for water treatment and stormwater management (Original data).
